# Supplementary material for: Complexome profiling on the Chlamydomonas lpa2 mutant reveals insights into PSII biogenesis and new PSII associated proteins
Source: J Exp Bot. 2021 Aug 26;73(1):245–62. doi: 10.1093/jxb/erab390 (PMC8730698; doi:10.1093/jxb/erab390)
Supplement: erab390_suppl_Supplementary_Dataset_S1 [file erab390_suppl_supplementary_dataset_s1.zip › Supplemental Dataset 1 - Excel List and all profiles/plots/DPS1_Cre02.g107300.html]

Trivial name: DPS1  
  
Euclidean distance: 3207.91  
Mean Intensity (WT): 160.60  
Mean Intensity (Mut): 216.66  
Distance: 14.81  
  
MapMan: amino acid metabolism.synthesis.aspartate family.lysine.dihydrodipicolinate synthase;amino acid metabolism.synthesis.aspartate family.lysine  
  
p value of intensity sums Welch test: 0.8226
